# Functioning of young patients with cerebral palsy: Rasch analysis of the pediatric evaluation of disability inventory computer adaptive test daily activity and mobility

**DOI:** 10.1186/s12955-020-01624-5

**Published:** 2020-11-18

**Authors:** Maíra Ferreira Amaral, Rosana Ferreira Sampaio, Wendy Jane Coster, Mariana Peixoto Souza, Marisa Cotta Mancini

**Affiliations:** 1 Graduate Program in Rehabilitation Sciences, School of Physical Education, Physical Therapy and Occupational Therapy, Universidade Federal de Minas Gerais (UFMG), Avenida Antônio Carlos, 6627, Pampulha, Belo Horizonte, MG 31270-010 Brazil; 2 Department of Occupational Therapy, Universidade Federal do Triângulo Mineiro (UFTM), Avenida Getúlio Guaritá, 159, Nossa Senhora da Abadia, Uberaba, MG 38025-440 Brazil; 3 Department of Occupational Therapy, College of Health and Rehabilitation Sciences: Sargent College, Boston University, 635 Commonwealth Avenue, Boston, MA 02215 USA

**Keywords:** Cerebral palsy, Activities of daily living, Mobility limitation

## Abstract

**Background:**

People with cerebral palsy experience limitations in performing activities of daily living. Rehabilitation practitioners seek valid instruments to measure changes in the performance of those activities. The Pediatric Evaluation of Disability Inventory Computer Adaptive Test (PEDI-CAT) is a new tool to assess functioning in children and youth with various health conditions. Its validity needs to be evaluated in a way that is consistent with the theoretical model on which it was based. We aimed to evaluate the fit of daily activity and mobility items and children with CP to the Rasch model and to compare the performance in daily activities and mobility of older children, adolescents, and young adults with CP based on manual function and gross motor function limitations.

**Methods:**

Eighty-three parents of children and youth of 8–20 years old (mean age: 11.6) with different severity levels of cerebral palsy participated in this study. Ninety-one items of the PEDI-CAT Daily Activities and Mobility domains were analyzed through Rasch analysis to evaluate relative item difficulty and participant ability. Participants were described according to the Manual Ability (MACS) (level I: 21.7%; II: 32.5%; III: 24.1%; IV: 7.2% and V: 3.6%) and the Gross Motor Function (GMFCS) (level I: 37.3%; II: 26.5%; III: 6%; IV: 18.1%; and V: 7.2%) classification systems levels.

**Results:**

Our data fit the Rasch Model. Parents had difficulty distinguishing some PEDI-CAT response categories. Participants from MACS and GMFCS levels IV and V showed lower ability to perform relatively more difficult items. There was a floor effect in both domains. Only 7.7% of the items presented differential item functioning when individuals with mild MACS and GMFCS levels (I, II) and moderate level (III) and individuals with moderate (III) and severe levels (IV, V) were compared.

**Conclusions:**

PEDI-CAT daily activities and mobility domains are valid to evaluate children, adolescents and youth with CP of different severities, but the addition of items to these domains is recommended in order to address their floor effect.

## Background

Children, adolescents and young adults with cerebral palsy (CP) experience limitations in performing activities of daily living [1]. Parents and their children with CP often identify goals from the levels of activities and participation [2–5].

Daily activities and mobility are important aspects of functioning which are essential to support children’s and adolescents’ participation in relevant contexts such as the school, home, and community [6]. Interventions focusing on daily activities and mobility for children, adolescents, and young adults with CP should be guided by measures that properly assess these outcomes [7, 8]. Rehabilitation practitioners seek instruments that can measure changes in the performance of relevant activities in children with CP. Such instruments must show good psychometric properties and be easy-to-use [9].

The Pediatric Evaluation of Disability Inventory Computer Adaptive Test (PEDI-CAT) [10] assesses functioning in children, adolescents and young adults with various health conditions. The PEDI-CAT is the new version of the Pediatric Evaluation of Disability Inventory (PEDI), which is frequently used as an outcome measure for children with CP [11–13]. The PEDI-CAT was developed using current methodologies for test construction, including Item Banking, Item Response Theory (IRT), and Computer Adaptive Testing (CAT). The items are computer-selected from the responses provided by the respondent based on algorithms [10]. This strategy yields improved test score precision and a reduction in assessment time. As a consequence, procedures to test its validity must acknowledge and be congruent with these methodologies.

As the original PEDI assesses functioning in children from 0 up to 7 years, it has a restricted follow-up window [14]. A major contribution of the revised version of PEDI, the PEDI-CAT, is its expanded age coverage from childhood to adolescence and early adulthood, up to 20 years old. Because of these changes, it is important to investigate the validity of PEDI-CAT for older children, adolescents, and youth with CP, whose age ranges were not covered by the original version.

Strong psychometric properties of the PEDI-CAT have been documented for individuals with and without disabilities [15–17]. Its application so far includes the evaluation of functioning in children and young people with autism spectrum disorders [18], complex medical conditions [19], HIV encephalopathy [20] and spinal muscular atrophy [21].

Investigation of the PEDI-CAT validity for children and youth with CP has used procedures from the Classical Test Theory (CTT). Frazier et al. [22] analyzed the relationship between PEDI-CAT domain scores and categories of CP type and severity. They found associations between CP type and severity with PEDI-CAT scores in all domains. Children with bilateral CP and those from the more severe category scored lower (raw and normative PEDI-CAT scores) than children from other categories. Shore et al. [23] examined two aspects of discriminant validity of the PEDI-CAT domains: (1) ability to differentiate the degree of independence in fine and gross motor function, using receiver operating characteristics (ROC) curve analysis, and (2) differentiation across all Gross Motor Function Classification System (GMFCS) and Manual Ability Classification System (MACS) levels, using general linear modeling. Their results showed that Mobility and Daily Activity domains were able to discriminate, respectively, all GMFCS and three of five MACS levels, whereas the other domains were able to discriminate fewer GMFCS and MACS functional levels. A recent longitudinal study by Burgess et al. [24] investigated self-care developmental trajectories from a population-based sample of children with cerebral palsy (CP), ages from 2 years and 6 months to 12 years of age, across all functional ability levels, according to the MACS classification system. Their results identified different PEDI-CAT DA scores for all five manual functions MACS levels, implying PEDI-CAT was able to distinguish different manual functional levels. To date, and to the best of our knowledge, no study has used Rasch analysis to evaluate the validity of the PEDI-CAT daily activity and mobility items in older children and youth with CP.

Rasch analysis is a probabilistic mathematical model derived from IRT, which transforms raw scores into linear continuous measures of a person’s ability and item difficulty. It allows people and items to be located along the same continuum and to be measured by the same unit, called logit [25]. This model is an alternative to traditional methods in psychometrics and has been widely used in recent studies evaluating the psychometric properties of different instruments [26]. Thus, the analysis of PEDI-CAT psychometric properties using the Rasch approach will further evaluate the validity of this instrument for children and youth with CP, and do so in a way that is consistent with the theoretical model on which it was based. This study aimed to (1) evaluate the fit of daily activity and mobility items and children with CP to the Rasch model, including scale dimensionality, item difficulty, and the reliability and adequacy of the response categories and (2) compare the performance in daily activities and mobility of older children, adolescents, and young adults with CP based on manual function and gross motor function limitations.

## Methods

### Study design

The data for this study were collected as part of a larger cross-sectional study on PEDI-CAT translation and cultural adaptation to the Brazilian Portuguese [27].

### Participants

Children with CP who were between 8 and 20 years of age and their parents or caregivers participated in this study. According to Linacre [28], a sample greater than 50 participants is sufficient to obtain stable item calibrations, considering, with 99% confidence, that no item calibration will have more than 1 logit of its stability value. The sample was recruited by convenience in rehabilitation institutions from the cities of Belo Horizonte and Fortaleza, Brazil. Participants were considered eligible for this study if: (1) they identified themselves as a parent or legal guardian of the child or youth; (2) their child or youth had medical diagnosis of CP of any level of severity; (3) their child or youth was between 8 and 20 years old at the time of enrollment.

### Instruments

#### Pediatric evaluation of disability inventory-computer adaptive test (PEDI-CAT)

The PEDI-CAT is a comprehensive functional assessment for children and young people with various health conditions that measures the following four domains: daily activities (DA), mobility (MB), social/cognition (SC), and responsibility (RS). The PEDI-CAT consists of a large item bank of 276 functional activities. In this study we used data collected for a previous study of translation and cultural adaptation of PEDI-CAT to the Portuguese language, Brazil [27]. At the time, the CAT wasn’t available. Thus, the instrument items were administered to the parents using paper and pen. Following instructions provided by the authors of the PEDI-CAT test, we conducted the distribution of the 276 PEDI-CAT items into age groups so that it would not be necessary to administer all items to the entire sample. The 276 PEDI-CAT items were then distributed to three age groups (0–7, 8–14, and 15–20 years of age) by experienced professionals in child development based on the age appropriateness of each item. For this study, we worked with 46 DA items, 23 MB items, and 22 mobility disability (MBD) items (for those who used a walking aid and/or wheelchair), totaling 91 items. These items were administered to participants aged 8–20 from the previous study. In both studies, all items were administered to all study participants.

DA items include activities related to getting dressed, keeping clean, home tasks, and eating and mealtime, (e.g. pours liquid from a large carton into a glass; Puts toothpaste on brush and brushes teeth thoroughly). MB items include activities related to basic movement and transfers, standing and walking, steps and inclines, running and playing (e.g., walks fast enough to cross two-lane street safely; Pulls self out of swimming pool not using ladder). MBD items consist of a section of the MB domain, including a subset of activities performed with walking aid and/or wheelchair (e.g. steps up and down curbs using walking aid—cane, crutches, walker). In these domains, scoring is based on a four-point ordinal scale with different levels of difficulty: (1) Unable, (2) Hard, (3) A little hard, and (4) Easy [10].

#### Gross motor function classification system (GMFCS) and manual ability classification system (MACS)

Professionals working with participants at the two rehabilitation clinics classified CP severity using GMFCS [29] and MACS [30]. These systems classify gross motor function and manual function, respectively, on levels from I to V (I: least severe; V: most severe).

### Data analyses

Rasch analyses verified the applicability of PEDI-CAT DA and MB items to older children, adolescents, and young adults with CP. The Rasch Rating Scale Model was used for this study because the PEDI-CAT response categories are polytomous. This model estimates (1) the functional parameter in daily activities and mobility of each individual with CP, (2) the difficulty of performing each item, (3) a “step” difficulty estimate associated with progressing from one response category to the next highest one (e.g., from “Unable” to “Hard”). The WINSTEPS® software package version 3.92.1 was used for data analysis.

Goodness-of-fit tests were performed for each domain (DA and MB) to indicate how well the data fit the Rasch Model. The analyses are based on mean square (MNSQ) and Z-standardized scores (ZSTD). OUTFIT MNSQ values between 0.5 and 1.5 are considered acceptable [31]. We chose OUTFIT values because it is more sensitive to outliers’ responses. When individual or item values do not fall within this range, the researcher must check ZSTD statistics. If > 2, the individual or item is considered a misfit. When a misfit is found, Linacre [32] suggests that individuals and/or items be withdrawn in descending order of misfit, and new analyses should be performed and new goodness-of-fit values should be checked. This process must be repeated until data fit the Rasch Model.

Reliability was assessed through person separation, person reliability, item separation, and item reliability indexes. These indexes reflect reproducibility (reliability indexes) and dispersion (separation indexes) of individuals and items along the continuum. Separation values > 2 and reliability values > 0.8 for both individuals and items, are considered reference values for these indexes [33]. If the item indexes are below the required values, a larger sample is necessary. Conversely, if the person indexes are below the required values, the test needs more items. We also provided the Chi-Square statistics values (*X*^2^). Non-significant *p* values (*p* > 0.01) reflected data homogeneity [30].

Principal component analysis (PCA) on the residuals of the Rasch model verified the unidimensionality of the DA and MB domains. According to the criterion adopted by Pasternak et al. [21], the amount of variance explained by the first dimension should be > 20% to consider the data to be one-dimensional.

Category characteristic curves (CCCs) examined the probability that each person will respond to each response category separately. They were developed for each item and for the set of items in each domain. Ludlow et al. [34] suggested that the ideal probability standard for these CCCs occurs when each successive response category is the most likely response at some point along the continuum of the person's ability. This probabilistic pattern occurs when the estimates of the response categories strictly follow an increasing order.

The item-person maps illustrate the calibration of individuals and items hierarchically, in order of relative difficulty, identifying more difficult and less difficult items and individuals with higher and lower abilities along the same one-dimensional continuum. For this study, to analyze DA items and person estimates, we defined each individual according to his/her MACS level. Similarly, for the MB domain, we described individuals by their GMFCS level. Analyses examined the Differential Item Functioning (DIF) of DA and MB for individuals with MACS and GMFCS mild levels (I, II) and moderate level (III) and for individuals with moderate (III) and severe levels (IV, V). According to Linacre [31] DIF should be considered significant if the DIF contrast statistic is > 0.5 logits (DIF contrast > 0.5) and t statistic is > 2.0 (t > 2.0).

## Results

### Descriptive data

Eighty-three (N = 83) parents or caregivers and their child/youth participated in this study (Table 1).

**Table 1:** Descriptive information of children and youth with cerebral palsy (n = 83)

Children/youth	Age (years)^a^		11.6 (2.9)
	Sex^b^	F	39 (47.0%)
		M	44 (53.0%)
Family socioeconomic information^bc^	Socioeconomic classification	A	2 (2.4%)
		B	22 (26.5%)
		C	46 (55.4%)
		D	12 (14.5%)
GMFCS^bc^		I	31 (37.3%)
		II	22 (26.5%)
		III	5 (6.0%)
		IV	15 (18.1%)
		V	6 (7.2%)
MACS^bc^		I	18 (21.7%)
		II	27 (32.5%)
		III	20 (24.1%)
		IV	6 (7.2%)
		V	3 (3.6%)

*F* female, *M* male; Brazilian socioeconomic classification, where *A* highest socioeconomic level, *D* lowest socioeconomic level, *GMFCS* gross motor function classification system and *MACS* manual ability classification system, where I and II mild levels, III moderate level, IV and V severe levels

^a^Mean (standard deviation)

^b^Count (percent)

^c^Numbers do not add up to N = 83 due to missing data

### Fit analyses

For the DA domain, the initial model with all individuals and items showed misfit for 3 individuals and for item DA040 (*Puts hair up in a ponytail*). We followed rules previously described, beginning with the withdrawal of misfit individuals. When the third person was removed from the model, only item DA068 (*Puts on slip-on shoes*) remained with MNSQ = 1.99 and ZSTD = 2.1, slightly above the reference values; one other individual showed misfit. After the withdrawal of these four individuals, the items and people fit the Rasch Model. The DA final model had 79 individuals and 46 DA items.

The initial MB domain model showed misfit for 5 individuals and 7 items. Individuals who showed misfit were removed. After each withdrawal, new individuals and items presented misfit. We removed 11 individuals and then, items MB120D (*Goes up and down curbs with wheelchair*) and MB071 (*Stands while holding on in a moving vehicle like bus, train, trolley, boat/ferry*) presented misfit and we decided to withdraw both items from the model. After these adjustments, individuals and items fit the Rasch model. The final MB model had 72 participants and 43 items.

### Reliability and homogeneity

Table 2 shows the reliability indexes considering the real non-extreme persons and items values in the sample (i.e., items or persons with 0% and 100% success rates), since the measures for extreme scores are imprecise [33]. The person separation index values indicated that the study sample could be divided into ability categories (e.g., individuals with higher and lower ability to perform DA and MB activities). The item separation index showed that the sample was large enough to identify the relative difficulty of PEDI-CAT items. *p* values for chi-square statistic were non-significant (*p* = 0.50 for DA and *p* = 0.39 for MB) indicating data homogeneity across the different class intervals.

**Table 2:** Reliability indexes (non-extreme persons and items) and homogeneity

PEDI-CAT domain	Person index		Item index		Log-likelihood	
	SEP	REL	SEP	REL	*X*^2^ (df)	*p* value
Daily activity	4.78	0.96	5.86	0.97	4556.49 (4558)	0.50
Mobility	6.94	0.98	7.61	0.98	2142.17 (2126)	0.39

*SEP* separation, *REL* reliability. Numbers represent reproducibility (reliability indexes) and dispersion (separation indexes) of individuals and items along the continua. Reference values for person and item: separation indexes > 2 and reliability indexes > 0.8. *X*^2^: Chi-square, *df* degrees of freedom. Reference values for log-likelihood: *p* > 0.01

### Unidimensionality

The PCA of the Rasch model residuals indicated that the amount of variance in the DA domain was 66.4% and for the MB domain was 76.8%. These results suggest that the DA and MB domains are unidimensional, that is, they measure a single construct.

### Category characteristic curves (CCCs)

**Fig. 1:**
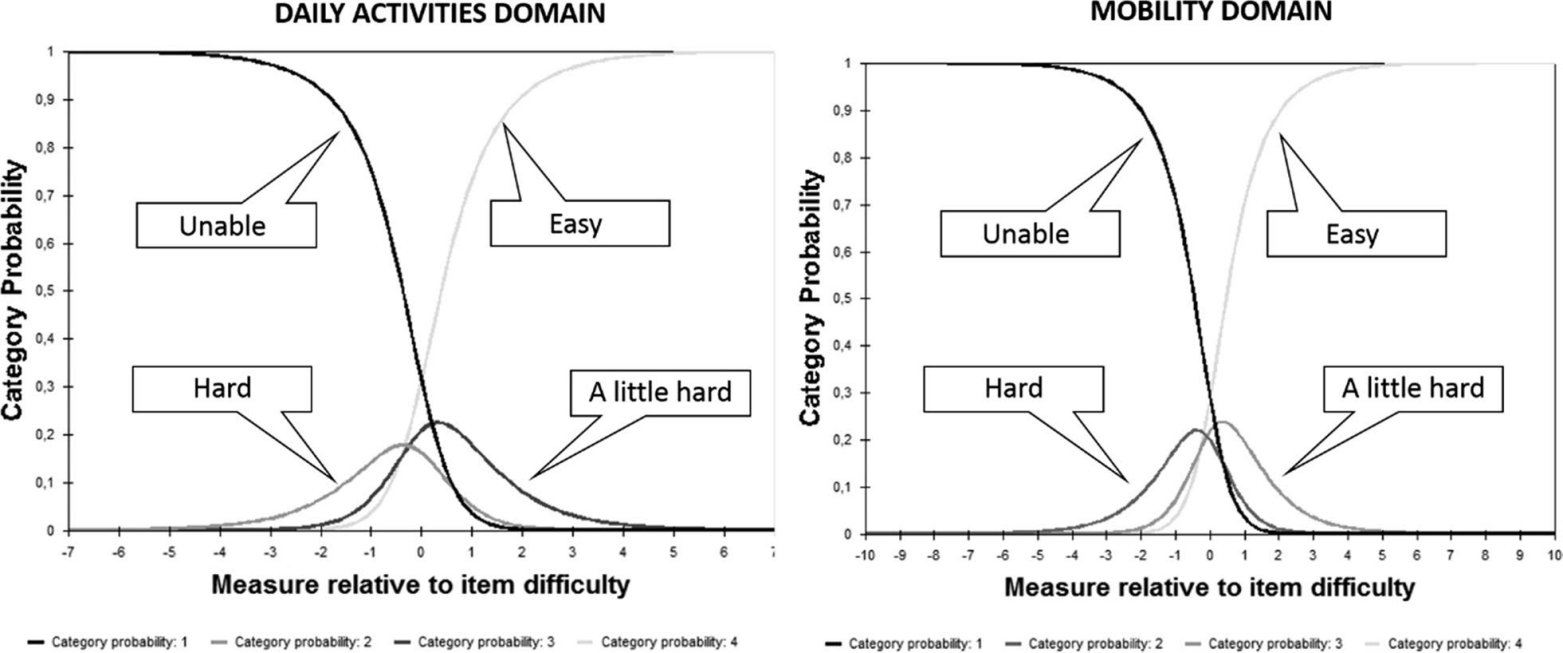
Category characteristic curves for daily activities and mobility domains. Legend: Curves represents the probability that each person will respond to each response category separately

Figure 1 shows the CCCs displayed by DA and MB domain. The curves show that the greatest probabilities of response lie at both ends of the response categories (*unable* and *easy*), while the middle categories have low occurrence probabilities (*hard* and *a little hard*).

### Item-person maps

**Fig. 2:**
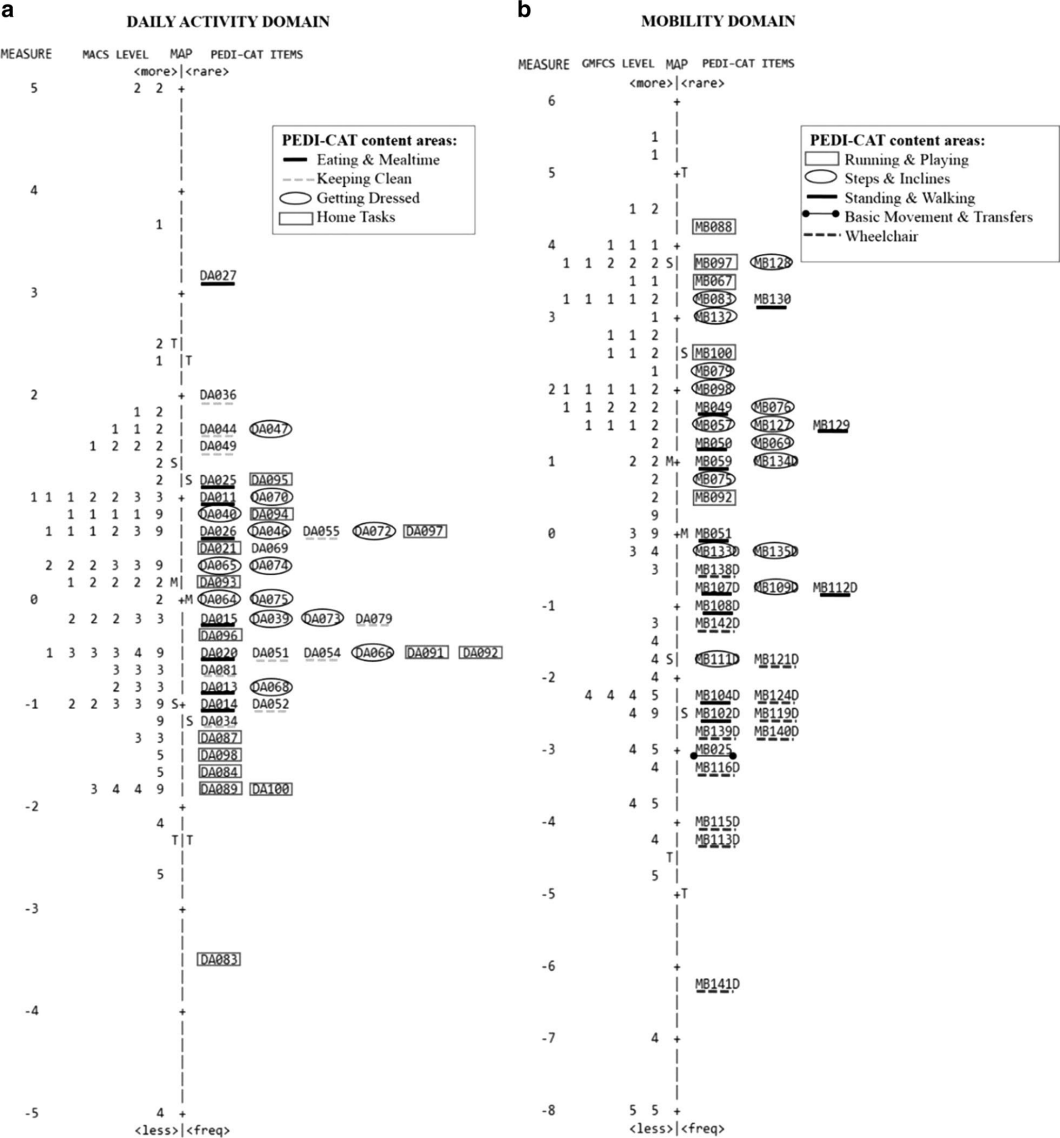
PEDI-CAT item-person maps from the final Rasch analyzes. Legend: **a** Daily activity items (items = 46) and persons (n = 79) classified according to MACS levels; **b** mobility items (items = 43) and persons (n = 72) classified according to GMFCS levels. 1, 2, 3, 4 and 5 refer to levels I, II, III, IV and V of the MACS and GMFCS classifications. 9 = missing data; M = Mean persons’ ability or mean items’ difficulty; S = one standard deviation; T = two standard deviations. The vertical line is a continuum representing the measures of persons’ ability (left side) and items’ difficulty (right side), plotted in logit units. The persons’ ability and items’ difficulty increase from bottom to the top

Figure 2a, b shows the item-person maps for the DA and MB domains, respectively. The numbers 1–5 correspond to the severity levels of CP classified by MACS (Fig. 2a) and the GMFCS (Fig. 2b). They begin from the lowest score (least functional) at the bottom to the highest score (most functional) at the top.

The 46 DA items are shown in Fig. 2a. Based on the item locations, it was easiest for participants with CP to perform DA083 (*Uses a TV remote control*) with no help, extra time or effort, followed by DA089 (*Wipes a counter or table*), and DA100 (*Removes a single bill from wallet*). These easy items are home tasks area. Most DA items are distributed in the middle of the continuum and address all areas of DA (getting dressed, keeping clean, home tasks, and eating and mealtime). The most difficult item to perform easily without help or extra time was DA027 (*Uses a can opener to open a can*), followed by DA036 (*Trims fingernails on both hands*), DA044 (*Shaves face using electric or safety razor*), DA047 (*Fastens a necklace or chain*), and DA049 (*Trims toenails on both feet*). These items are related to keeping clean.

On the left side of the map, participants with severe manual function limitation (MACS IV and V) are mostly located on the bottom, while those with mild limitation (MACS I and II) are at the top. Participants with moderate limitation (MACS III) are distributed in the middle. Some exceptions can be observed; some participants with mild CP are seen at the middle of the map.

The 43 mobility items distributed in the item-person map are shown in Fig. 2b. Based on the item locations, it was easiest for participants with CP to perform MB141D (*Puts wheelchair brakes on and off*) with no help, extra time or effort, followed by MB113D (*Uses wheelchair to move from room to room in home*), and MB115D (*Keeps place in a line of moving people while using wheelchair*). All disability items are located at the bottom and middle of the map, paired in position with participants with severe gross motor limitation. Item MB025 (*Gets under sheet or blanket and arranges pillows for comfort in bed*) was the easiest activity for those who do not use walking devices and it is also located at the bottom of the map. It was followed by MB051 (*Pushes adult-size shopping cart*), MB092 (*Pumps legs and swings on playground swing*), and MB075 (*Goes up and down an escalator*), which are located at the middle of the map. The most difficult mobility items to perform easily without help or extra time were MB088 (*Jumps 10 times in a row with a jump rope*), followed by MB097 (*Moves across monkey bars*), and MB128 (*Climbs step ladder to put a heavy box on a high shelf*). Most running and playing items are located at the top of the continuum, indicating that these items were difficult to perform.

Participants with severe gross motor function limitation are clustered mostly on the bottom, while those with mild limitations are at the top. Participants with moderate limitations are mostly distributed in the middle of the continuum. Participants classified as GMFCS III, IV, and V had logit values similar to the disability mobility items.

We noted some inversions of CP severity levels along the functional domains’ continua. For example, in both DA and MB domains, we observed individuals with level IV of motor severity in a higher position than others with level III. Likewise, some level V individuals appeared in positions higher than level IV individuals, and some level II individuals of motor severity presented greater ability than level I individuals. Some gaps among the items at both ends of the DA continuum and at the bottom of the MB continuum are observed.

Three DA and two MB items showed DIF when mild and moderate motor function limitation levels were compared (DA073: *Puts on winter, sport, or work gloves*; DA091: *Stacks breakable plates or cups*; DA093: *Changes pillow case on pillow*; MB 097: *Moves across monkey bars;* MB100: *Pulls self out of swimming pool not using ladder*). DA095 (*Tightens loose screws using a screwdriver*) and MB025 (*Gets under sheet or blanket and arranges pillows for comfort in bed*) demonstrated DIF when moderate and severe motor functional limitation levels were compared.

## Discussion

This study investigated the validity of PEDI-CAT using Rasch analysis to verify if the DA and MB domains of this instrument are suitable to evaluate the functioning of children and youth with CP. The results showed that 46 items of DA and 43 items of MB fit the Rasch Model. Reliability and PCA scores point to a good fit to the Rasch model, which is consistent with previous studies that evaluated the PEDI-CAT in IRT-based models with other clinical groups [10, 35].

The CCCs did not show the expected pattern for the PEDI-CAT response categories. 36.41% of responses of the DA domain scored 1 (Unable) and 43.99% scored 4 (Easy). For the MB domain, 49.44% of responses scored 1 (Unable) and 33.07% scored 4 (Easy). It suggests that parents or caregivers of children and youth with CP had difficulty distinguishing some response categories (i.e., the medium response categories of hard and a little hard). More frequently, parents had a tendency to choose the two extreme and opposing categories of response (e.g., unable and easy) when answering the PEDI-CAT items. Pasternak et al. [21], found similar results and suggested that, in future reviews of the instrument, the “hard” and “a little hard” response categories should be collapsed. Our results suggest that the middle response categories, which describe the process of skill acquisition, do not seem to be considered by the parents and caregivers when scoring the DA ad MB items. Parents seemed to base their evaluation of their children’s levels of ability as the activities that they can or cannot perform and partial performance or performance with effort were not recognized. In contrast, rehabilitation professionals are often interested in measuring and documenting intermediate stages of the skill acquisition process, when children evolve from being unable to perform an activity to being able to perform only part of an activity, sometimes with a lot of effort, to further being able to perform most of the activity and finally performing this activity with no difficulty. Further studies should verify that parents understand all possibilities of the scoring criteria of the new PEDI-CAT.

The item-person maps showed some inversions of CP severity levels along the two continua, especially in the DA domain. This result suggests that performance of daily activities and mobility activities [1, 23] is not directly determined only by impairment on the gross motor and manual function but, rather, by a combination of factors, including other body functions (e.g., cognitive functions), and personal and environmental factors [36]. It is possible, for example, that a child with CP who has a moderate level of motor function limitation may have environmental supports that allow him/her to create strategies to perform activities, in contrast to another child with a mild level of limitations who does not have the same supports. In that sense, it is possible that children with better manual skills (i.e. MACS I and II) who have cognitive deficits may show difficulty performing more complex daily activities (e.g. *Ties shoelaces*). Also, studies on prognoses of gross motor function and manual skill have shown that there is some instability of the GMFCS and MACS classification levels over time, that is, children classified as MACS level II, e.g. undergoing intensive rehabilitation, may further become MACS level I [37, 38].

These inversions could also explain the DIF results, which demonstrated that children and youth with a moderate CP level of manual function were able to perform item DA073 (*Puts on winter, sport, or work gloves*) more easily than children with a mild CP MACS level. These results highlight the multifactorial nature of functioning, emphasizing the person-environment interaction as the relevant unit of analysis in functional assessment.

Only five items showed DIF between people with mild and moderate motor function limitations and other two items revealed differences between individuals from moderate and severe GMFCS level. In the DA domain, the three items that differentiated mild and moderate severity levels were complex items from Dressing and Home Task areas. In the MB domain, differences between these severity levels were revealed by two items from the Running and Playing area, which require upper body strength (*Moves across monkey bars*; *Pulls self out of swimming pool not using ladder*). The number of items that presented DIF corresponds to 7.7% of the total items evaluated in this study. Coster et al. [35] found similar number of items in the DA domain of the PEDI-CAT that showed differential item functioning among children and youth with and without autism. The authors concluded that such small percentage required no modification in the PEDI-CAT parameters. However, studies have pointed out the need to, once DIF is found, use complementary methods to (1) analyze the magnitude of DIF, (2) analyze whether the magnitude of DIF results in an important clinical impact, considering the instrument as a whole, and (3) use analysis based on qualitative methods to interpret the reasons for the differential functioning of the item (i.e., conduct groups with area experts) [39, 40]. Thus, further studies are needed to verify in greater detail the reasons that led parents of children and youth with different severity levels of CP to respond differently to these items.

Although most items are located in the same region as most individuals, at the extremes of both continua there are some participants who are not aligned with any item. This suggests that the difficulty of the items is either too low or too high for the ability levels of some participants and that it may be necessary to add very easy items and very difficult items in both domains. In addition, the results showed that most items in the Running and Playing area are located at the top of the continuum. It might be useful, then, to develop other items in this area with lower levels of difficulty. This addition of items could help to better identify the limitations in performance in daily activities and mobility of children and youth with CP with mild and severe levels of motor function as recently also recommended by Burgess et al. [24]. One great advantage of instruments that use the CAT system is the possibility of adding new items to the item bank, thus allowing the instrument to become more complete and appropriate as results from new validation studies may indicate [41, 42].

Most DA items located at the bottom of the item-person map refer to the use of electronic devices such as the remote control, phone, and computer. The easiest MB items were simple tasks involving wheelchair management. This is not surprising as these activities involve minimal physical effort and allow the use of compensatory strategies to manage assistive technology devices [43–45]. Conversely, the most difficult DA items were those that required fine bimanual coordination. In this study, most participants with mild manual function limitation had spastic hemiplegic CP type, which is characterized by a unilateral upper and lower extremity involvement. This may explain the difficulty encountered by these mild severity level participants in performing such bimanual activities [46].

PEDI-CAT items for individuals who use mobility devices (such as cane, crutches, walker) and wheelchair were consistent with participants’ GMFCS levels. In this classification system, people who make use of walking devices are classified in levels III, IV, or V of gross motor function. In the item-person MB map, disability items are located throughout the continuum of difficulty, and paired with participants classified as GMFCS levels III, IV, and V.

This study demonstrated that DA and MB PEDI-CAT items are able to discriminate individuals with CP of different functional levels, as we can observe through the location of individuals and items on the two ability/difficulty continua (Fig. 2). These results are in accordance with those reported by Frazier et al. [22] and Shore et al. [23], who used CTT-based analyzes to verify the validity of PEDI-CAT in discriminating functional levels of children with CP. Analyzes conducted by those studies compared GMFCS and MACS categories according to scores obtained by the participants in each PEDI-CAT domain. However, they were not able to identify which items or functional areas within the PEDI-CAT domains are more suitable for specific CP levels. By focusing on items and individuals and their relative difficulties using Rasch analysis, our study moves the validity investigation of the PEDI-CAT forward. This study had a small number of participants, especially older ones (e.g., adolescents and young adults) and with severe and moderate motor function limitations, which may limit the generalization of our results. We also acknowledge that the decision to curb the PEDI-CAT item pool represents a limitation of the present study, that needs to be addressed in future studies (Additional file 1).

## Conclusions

Our results provide evidence that the PEDI-CAT DA and MB domains are valid to evaluate older children, adolescents and young adults with CP. We observed that parents had a tendency to choose between the two extreme and opposing PEDI-CAT response categories. There was a floor effect in both domains, that is, the difficulty level of some items was either too low or too high to measure the CP individuals’ abilities. It is recommended to add very easy items and very difficult items to the DA and MB domains, especially easiest items in the Running and Playing content area. Only few items presented DIF, indicating that the DA and MB domains are valid to evaluate CP participants with different manual abilities and gross motor function levels. Understanding the performance profile of older children, adolescents, and young adults with CP in daily activities and mobility, by focusing on items’ difficulties and individuals’ abilities support more appropriate evaluation and interventions for this population.

## Supplementary information

**Additional file 1.** List of PEDI-CAT Daily Activities and Mobility items and indication of their use in the data analysis.

## Data Availability

The datasets used and/or analyzed during the current study are available from the corresponding author on reasonable request.

## Abbreviations

PEDI-CAT: Pediatric Evaluation of Disability Inventory Computer Adaptive Test
PEDI: Pediatric Evaluation of Disability Inventory
CP: Cerebral palsy
MACS: Manual ability classification system
GMFCS: Gross motor classification system
IRT: Item response theory
CAT: Computer adaptive testing
CTT: Classical test theory
DA: Daily activities
MB: Mobility
SC: Social/cognition
RS: Responsibility
MBD: Mobility disability
MNSQ: Mean square
ZSTD: Z-standardized
PCA: Principal component analysis
CCCs: Category characteristic curves
DIF: Differential item functioning
