# Supplementary material for: Functioning of young patients with cerebral palsy: Rasch analysis of the pediatric evaluation of disability inventory computer adaptive test daily activity and mobility
Source: Health Qual Life Outcomes. 2020 Nov 18;18:369. doi: 10.1186/s12955-020-01624-5 (PMC7672984; doi:10.1186/s12955-020-01624-5)
Supplement: Supplementary file 1 — Additional file 1. List of PEDI-CAT Daily Activities and Mobility items and indication of their use in the data analysis. [file 12955_2020_1624_MOESM1_ESM.docx]

**Supplementary Table**: The PEDI-CAT Daily Activities and Mobility Items Used and Not Used in the Rasch Analysis

| **DAILY ACTIVITIES** | **Used**/ **Not Used** | **Reason for not using*** |
| --- | --- | --- |
| Swallows pureed/ blended/ strained foods | Not Used | Age |
| Finger feeds small or bite-size pieces of food | Not Used | Age |
| Holds and drinks from an open cup or glass | Not Used | Age |
| Holds and eats a sandwich or burger | Not Used | Age |
| Feeds self with spoon (minimal spilling) | Not Used | Age |
| Drinks liquids using a straw | Not Used | Age |
| Feeds self with fork (minimal spilling) | Not Used | Age |
| Uses a knife to butter bread and spread jam | Not Used | Age |
| Inserts a straw into a juice box | Not Used | Age |
| Pulls open a sealed bag of snack food | Not Used | Age |
| Removes lid from plastic food containers | Not Used | Age |
| Closes a bottle with a twist-off cap | Not Used | Age |
| Rubs hands together to clean | Not Used | Age |
| Wipes nose thoroughly with tissue | Not Used | Age |
| Turns the water on and off at sink | Not Used | Age |
| Removes socks | Not Used | Age |
| Takes off a t-shirt | Not Used | Age |
| Puts on a t-shirt | Not Used | Age |
| Puts on and buttons a front-buttoning shirt | Not Used | Age |
| Removes pants with elastic waist | Not Used | Age |
| Puts on socks | Not Used | Age |
| Uses a computer mouse to click on icons or links | Not Used | Age |
| Cuts vegetables or meat with a fork and table knife | Used |  |
| Pours liquid from a large carton into a glass | Used |  |
| Stirs to mix ingredients | Used |  |
| Empties food from mixing bowl to baking pan | Used |  |
| Opens sealed cardboard food boxes | Used |  |
| Cuts with scissors to open hard plastic packaging | Used |  |
| Peels foods such as potatoes or carrots | Used |  |
| Chops or slices hard fruits or vegetables | Used |  |
| Uses a can opener to open a can | Used |  |
| Puts toothpaste on brush and brushes teeth thoroughly | Used |  |
| Trims fingernails on both hands | Used |  |
| Fastens hairclips or barrettes | Used |  |
| Puts hair up in a ponytail | Used |  |
| Shaves face using electric or safety razor | Used |  |
| Fastens watch band | Used |  |
| Fastens a necklace or chain | Used |  |
| Trims toenails on both feet | Used |  |
| Cleans body thoroughly in bath or shower | Used |  |
| Dries hair with a towel | Used |  |
| Obtains shampoo, washes and rinses hair | Used |  |
| Dries hair with a hair dryer | Used |  |
| Puts on and fastens pants | Used |  |
| Fastens belt buckle | Used |  |
| Tucks in shirt or blouse | Used |  |
| Puts on slip-on shoes | Used |  |
| Connects and zips zippers that are not fastened at the bottom | Used |  |
| Ties shoelaces | Used |  |
| Inserts laces into sneakers or boots | Used |  |
| Puts on winter, sport, or work gloves | Used |  |
| Puts on bra and fastens in front or back | Used |  |
| Puts on tights or pantyhose | Used |  |
| Wipes self with toilet paper after a bowel movement | Used |  |
| Opens, closes and latches public bathroom stall doors | Used |  |
| Uses a TV remote control | Used |  |
| Operates a video game controller | Used |  |
| Uses a computer keyboard to type | Used |  |
| Wipes a counter or table | Used |  |
| Stacks breakable plates or cups | Used |  |
| Opens door lock using key | Used |  |
| Changes pillow case on pillow | Used |  |
| Replaces (unscrews and screws) the bulb in a table lamp | Used |  |
| Tightens loose screws using a screwdriver | Used |  |
| Puts a bandage on a small cut on hand | Used |  |
| Opens childproof medicine or vitamin containers | Used |  |
| Presses buttons to operate a key-pad such as phone or ATM | Used |  |
| Removes a single bill from wallet | Used |  |
| **MOBILITY ITEMS** | **Used**/ **Not Used** | **Reason for not using*** |
| When lying on belly, turns head to both sides | Not Used | Age |
| When lying on back, turns head to both sides | Not Used | Age |
| When lying on back, reaches for toy | Not Used | Age |
| When lying on belly, pushes up on elbows | Not Used | Age |
| When lying on belly, pushes up on hands | Not Used | Age |
| Gets onto hands and knees | Not Used | Age |
| Sits on floor with pillow for support | Not Used | Age |
| Lifts one arm overhead and reaches for a small toy while sitting on floor | Not Used | Age |
| Sits on floor without support of pillow or couch | Not Used | Age |
| Stands up from an adult-size chair | Not Used | Age |
| Sits in an adult-size chair with a back | Not Used | Age |
| Rolls over in bed or crib | Not Used | Age |
| Gets in and out of own bed | Not Used | Age |
| Stands up from the middle of the floor | Not Used | Age |
| Stands for a few minutes | Not Used | Age |
| Stands on tiptoes to reach for something | Not Used | Age |
| Gets in and out of bathtub | Not Used | Age |
| Steps in and out of shower stall | Not Used | Age |
| Gets on and off an adult-sized toilet | Not Used | Age |
| While standing, bends over and picks up something from the floor | Not Used | Age |
| Squats down and then stands back up | Not Used | Age |
| Walks while holding onto furniture or walls | Not Used | Age |
| Walks from room to room in home (no stairs) | Not Used | Age |
| Walks in between a row of auditorium or movie theater seats | Not Used | Age |
| Opens and closes door to enter and exit home | Not Used | Age |
| Walks on wet, indoor slippery surfaces | Not Used | Age |
| Walks and carries a full glass without spilling | Not Used | Age |
| Walks while wearing a light backpack | Not Used | Age |
| Walks while wearing a heavy backpack | Not Used | Age |
| Pulls heavy wagon filled with toys or small child | Not Used | Age |
| Walks outdoors on grass, mulch or gravel | Not Used | Age |
| Steps up and down curbs | Not Used | Age |
| Walks on a raised narrow surface (curb/low wall) | Not Used | Age |
| Walks up and down ramp | Not Used | Age |
| Walks several hours at family or school outing such as zoo, amusement park or fair | Not Used | Age |
| When running, is able to go around people and objects | Not Used | Age |
| Moves forward on ride-on toys without pedals | Not Used | Age |
| Rides tricycle | Not Used | Age |
| Gets in and out of a car | Not Used | Age |
| Gets in and out of van, truck or sport utility vehicle | Not Used | Age |
| Goes up and down stairs by crawling or scooting on bottom | Not Used | Age |
| Walks up a flight of stairs without holding onto handrail | Not Used | Age |
| Walks up a flight of stairs holding onto handrail | Not Used | Age |
| Walks down a flight of stairs holding onto handrail | Not Used | Age |
| Kicks a rolling ball while standing | Not Used | Age |
| Jumps down off a single step | Not Used | Age |
| Sits on infant playground swing while swing is pushed | Not Used | Age |
| Climbs up ladder of a slide | Not Used | Age |
| Climbs on and off a climbing structure | Not Used | Age |
| Climbs out of swimming pool using pool ladder | Not Used | Age |
| Climbs onto couch or adult-size chair | Not Used | Age |
| When walking, is able to go around people and objects | Not Used | Age |
| Stands while holding on in a moving vehicle (bus, train, trolley, boat/ferry) | Not Used | Misfit |
| Goes up and down curbs with wheelchair | Not Used | Misfit |
| Gets under sheet or blanket and arranges pillows for comfort in bed | Used |  |
| Walks and carries a food tray | Used |  |
| Walks and carries a full shopping bag with handles | Used |  |
| Pushes adult-size shopping cart | Used |  |
| Hikes up hill 2-3 miles/3-5 kilometers | Used |  |
| Walks fast enough to cross two-lane street safely | Used |  |
| Rides bicycle without training wheels | Used |  |
| Gets on and off a public bus or school bus | Used |  |
| Goes up and down an escalator | Used |  |
| Walks up and down bleacher steps in gym or stadium | Used |  |
| Walks down a flight of stairs without holding onto handrail | Used |  |
| Carries full laundry basket with 2 hands up a flight of stairs | Used |  |
| Jumps 10 times in a row with a jump rope | Used |  |
| Pumps legs and swings on playground swing | Used |  |
| Moves across monkey bars | Used |  |
| Climbs indoor step ladder | Used |  |
| Pulls self out of swimming pool not using ladder | Used |  |
| Climbs over 2 foot high obstacle such as a baby gate | Used |  |
| Climbs step ladder to put a heavy box on a high shelf | Used |  |
| Walks 3 miles/5 kilometers | Used |  |
| Walks 50 feet/15 meters while carrying 25 pound/11 kilogram bag | Used |  |
| Runs up 2 flights of stairs | Used |  |
| Walks with walking aid (e.g. cane, crutches, walker) from room to room in home (no stairs) | Used |  |
| Walking with walking aid (e.g. cane, crutches, walker), keeps place in a line of moving people | Used |  |
| Walks with walking aid (e.g. cane, crutches, walker) on wet, indoor slippery surfaces | Used |  |
| Walks with walking aid (e.g. cane, crutches, walker) on grass, mulch or gravel | Used |  |
| Steps up and down curbs using walking aid (e.g. cane, crutches, walker) | Used |  |
| Walks with walking aid (e.g. cane, crutches, walker) up and down ramp | Used |  |
| Walks with walking aid (e.g. cane, crutches, walker) several hours at family or school outing such as zoo, amusement park or fair | Used |  |
| Uses wheelchair to move from room to room in home | Used |  |
| Keeps place in a line of moving people while using wheelchair | Used |  |
| Opens and closes door to enter and exit home while using wheelchair | Used |  |
| Uses wheelchair outdoors on grass, mulch or gravel | Used |  |
| Goes up and down ramp with wheelchair | Used |  |
| Pushes wheelchair for several hours at family or school outing such as zoo, amusement park or fair | Used |  |
| Walks up a flight of stairs with a walking aid (e.g. cane, crutches, walker) | Used |  |
| Using walking aid (e.g. cane, crutches, walker), gets on and off a public bus or school bus | Used |  |
| Walks down a flight of stairs with walking aid (e.g. cane, crutches, walker) | Used |  |
| Moves from wheelchair to adult size chair | Used |  |
| Uses wheelchair to move quickly indoors to answer a telephone or doorbell | Used |  |
| Fastens wheelchair seat belt | Used |  |
| Puts wheelchair brakes on and off | Used |  |
| Gets into wheelchair from floor | Used |  |

*Reasons for not including the item in the analysis:

Age: Because all parents needed to respond to every item in both domains, in order to minimize the burden on them we limited our item pool to those items most likely to be relevant for the age range represented by our sample (8-20 years old). Haley et al.^15^ followed a similar process during the standardization study.

Misfit: Individual or item OUTFIT MNSQ values below 0.5 and above 1.5 and ZSTD values above 2.
